# *ALK* FISH patterns and the detection of *ALK* fusions by next generation sequencing in lung adenocarcinoma

**DOI:** 10.18632/oncotarget.12705

**Published:** 2016-10-17

**Authors:** Sanja Dacic, Liza C. Villaruz, Shira Abberbock, Alyssa Mahaffey, Pimpin Incharoen, Marina N. Nikiforova

**Affiliations:** ^1^ University of Pittsburgh Medical Center, Department of Pathology, Pittsburgh, PA, USA; ^2^ University of Pittsburgh Cancer Institute, Pittsburgh, PA, USA

**Keywords:** ALK, immunohistochemistry, FISH, NGS, lung

## Abstract

Break-apart *ALK* FISH probe is the FDA approved approach for detection of *ALK* rearrangements in lung carcinoma patients who may benefit from ALK kinase inhibitors. The FISH assay can be technically challenging and difficult to interpret. ALK immunohistochemistry and next generation sequencing have been proposed as alternative approaches. In this study, we compared various *ALK* –FISH patterns to next –generation sequencing (NGS) for gene fusion detection, ALK immunohistochemistry (IHC) and tumor responses to crizotinib. 72 (4%) of 2116 lung adenocarcinoma were positive by *ALK-* FISH. Of 28 *ALK*-FISH positive cases selected for the study, FISH patterns included 15 (54%) cases with split signal, 10 (36%) with single orange signal and 3 (10%) with “mixed pattern”. 12 (80%) cases with split signal and 4 (40%) cases with single orange signal were positive by NGS and IHC, while mixed cases were all negative. Mutation analysis of discordant cases revealed multiple mutations including oncogenic mutations in *EGFR*, *KRAS*, *BRAF* and *ATM* genes. All discordant cases in groups with split and mixed signal showed a lower number of cells with rearrangement (mean 28.5%; range 20.5-36.9%). No statistically significant association between response to crizotinib and FISH patterns was observed (p=0.73). In contrast, NGS fusion positive cases were associated with more responses to crizotinib than NGS negative cases (p= 0.016). Our study suggests that *ALK* FISH alone may not be the most reliable assay for detection of *ALK* gene rearrangements, and probably should be used in parallel with ALK IHC and NGS for detection of gene fusions and mutations.

## INTRODUCTION

Rearrangements of the *ALK* gene occur in up to 5% of non-small cell lung carcinoma (NSCLC), and are associated with an objective response rate of about 65% in patients treated with the ALK inhibitor crizotinib [1–3]. The Vysis LSI *ALK* break apart FISH probe kit (Abbott Molecular) was used to identify patients with *ALK* rearrangement positive NSCLC in the first clinical trials, and therefore the US Food and Drug Administration (FDA) approved this commercially available assay as a companion diagnostics for detection of *ALK* rearrangements [1]. The assay is considered to be positive for *ALK* rearrangement if at least 15% of tumor cells show rearrangement. *ALK* FISH assay can be challenging due to technical difficulties requiring repeat testing, borderline cut off values, false positive and false negative results. Different assay approaches other than FISH have been proposed for identification of *ALK* rearrangement in lung carcinoma [4–10]. The results of ALK immunohistochemistry and its correlation with FISH have been extensively reported in the literature [4, 6, 11–17]. It has been shown that the ALK fusion protein in NSCLC can be difficult to detect with the ALK1 antibody, which is used to diagnose anaplastic large cell lymphoma [18]. Many technical modifications including antigen retrieval and the development of new antibodies have been reported to increase the overall performance of immunohistochemistry in the detection of *ALK* rearrangement. As a result some antibody clones (5A4, D5F3) demonstrated a sensitivity and specificity of 95-100% when compared to FISH [4, 19, 20]. Overall, strong staining seems to be specific for *ALK* rearrangement and therefore ALK IHC was suggested as a cost effective screening method [10, 21]. Furthermore, studies showed that positive ALK protein expression correlates with tumor response to ALK inhibitors [22]. Recently, Wiesner T. el al. identified a novel *ALK* transcript, *ALK*^ATI^, which arises independently of genomic aberrations at the *ALK* locus through alternative transcription initiation and which can be detected by ALK IHC [23]. Preliminary data showed that the patients with *ALK*^ATI^ may benefit from ALK inhibitors and authors suggested immunohistochemistry as a screening method [23].

RT-PCR was another approach to *ALK* assessment and is not recommended as an alternative to FISH testing [8, 24]. The existence of many variants of *EML4-ALK* and more recently reported fusions of *ALK* to *TGF* and *KIF5B* raised the possibility of additional variant fusions making multiplexed RT-PCR assays very difficult to optimize for clinical use [3, 25–28]. However, recent developments in next-generation sequencing (NGS) of DNA and RNA have created a new opportunity for simultaneous detection of a large number of gene fusions with known and unknown partner genes and parallel detection of gene mutations [10, 29–31]. The results of successful screening for oncogenic fusions by a highly multiplexed PCR amplicon-based targeted next generation sequencing method have been recently reported [29]. It can be used for detection of known and novel ALK fusions in formalin-fixed paraffin embedded (FFPE) tissue specimens and requires minimal input of RNA. In this study, we evaluated the detection of *ALK* gene fusions by a targeted NGS approach and compared the results with various *ALK*-FISH patterns, ALK IHC and response to crizotinib.

## RESULTS

### Patient characteristics

Demographic characteristic of 28 patients with FISH positive for *ALK* rearrangements were summarized in Table 1. There were no statistically significant associations between FISH patterns and patient age, gender and smoking history.

**Table 1:** Demographic and clinical characteristics of 28 *ALK* FISH positive patients

PATIENT CHARACTERISTICS	ALK FISH PATTERN			*P* VALUE
	SPLIT (n=15)	SINGLE ORANGE (n=10)	MIXED (n=3)	
Age at diagnosis (years)	59	71	71	
Median (range)	(42-74)	(49-79)	(36-79)	0.09
Gender (%)				
Female	7 (47)	3 (30)	0	0.35
Male	8 (53)	7 (70)	3 (100)	
Smoking history (%)				
Current	1 (7)	1 (10)	0	0.66
Former	8 (53)	7 (70)	3 (100)	
Never	6 (40)	2 (20)	0	
Stage at diagnosis (%)				
I	2 (13)	1 (10)	0	
II	0	1 (10)	0	0.71
IV	13 (87)	8 (80)	3 (100)	

### FISH results

Of 28 *ALK*-FISH positive cases FISH patterns included 15 (54%) cases with fusion and split signal, 10 (36%) cases with single orange and 3 (10%) with a combination of fusion with split and single orange signals (“mixed pattern”). The average number of analyzed tumor cells was 75 (range 61-140). The average percentage of *ALK* positive cells in the fusion and split signal group was 57.3% (range 20.5%-92.5%), in the fusion and single orange signal group 77.8% (range 50%-90.4%) and in the mixed pattern group 24.6% (range 20.7%-30.5%).

### Next generation sequencing

**Figure 1:**
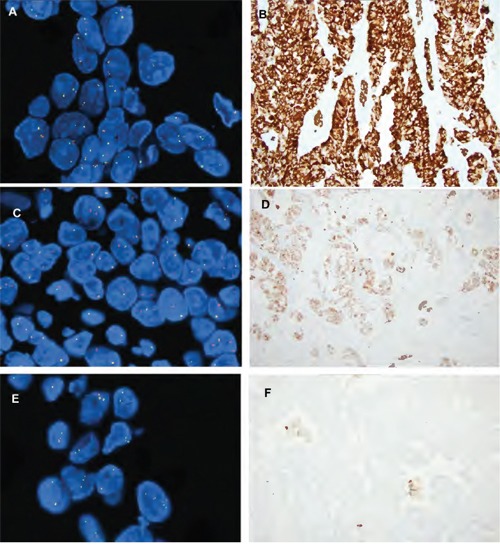
Examples of ALK FISH patterns and ALK IHC **A.** ALK FISH fusion and split signal with **D.** corresponding strong ALK IHC staining (magnification 20x); **B.** ALK FISH single orange signal and **E.** corresponding ALK IHC weak staining (magnification 20x). **C.** ALK FISH split pattern with **F.** ALK IHC negative staining (magnification 20x).

The results of fusion detection by NGS and by FISH are summarized in Table 2. Sixteen *ALK* fusions were detected by targeted NGS analysis. The most common fusion type was *EML4* exon 13 and *ALK* exon 20 detected in 8 of 16 (50%) cases, followed by *EML4* exon 6 and *ALK* exon 20 (3/16 (19%)), and one case was positive for *EML4* exon 20 and *ALK* exon 20 fusion. Four cases did not show reads mapped to the known fusion types, but instead demonstrated a strong differential expression between TK (3′-end) and EC (5'-end) domains of *ALK*. Such pattern indicates *ALK* fusion either with novel partner or with unknown breakpoint. The largest number of NGS positive cases (80%) was detected in the FISH group with split signal with *EML4-ALK* fusions detected in 10 of 15 cases (67%). Nine of those cases were available for immunohistochemistry and they were all positive (Figure 1). Two additional cases were negative for fusions, but showed an elevated 3′/5'*ALK* ratio. One of those two cases was positive by immunohistochemistry, while the IHC negative case demonstrated heterozygous loss of the *ALK* gene, *KRAS* G12V mutation and loss of *CDKN2A* (Figure 1). In that case FISH demonstrated only 27.6% of tumor cells with fusion and split signal. Three cases with FISH split signal were negative by NGS, and 2 of 3 cases that had sufficient tumor tissue were negative by immunohistochemistry. The average number of tumor cells with split signal in those cases was 27.5% (range 20.5-36.9%). Mutation analysis revealed *KRAS* G12V mutation in one case, while the second case showed mutation in *ATM* gene (Table 3).

**Table 2:** Summary of NGS *ALK* fusion detection and FISH patterns

FISH PATTERN	NEXT GENERATION SEQUENCING			
	POSITIVE FOR *EML4-ALK* FUSION	POSITIVE FOR *ALK* FUSION BY 3′/5' READ RATIO ONLY	NEGATIVE	TOTAL POSITIVE
**Split signal (N=15)**	10 (67%)	2 (13%)	3 (20%)	12 (80%)
**Single orange** **(N=10)**	2 (20%)	2 (20%)	6 (60%)	4 (40%)
**Mixed pattern (N=3)**	0 (0%)	0 (0%)	3 (100%)	0 (0%)

**Table 3:** Summary of mutation analysis of NGS *ALK* fusion negative cases

FISH	Case #	FISH POSITIVE CELLS (%)	IHC	GENOMIC CHANGES	
				MUTATIONS (% Mutant Allele Frequency)	COPY NUMBER CHANGES
**Fusion and split signal** **(N=3)**	1	20.5	NEG	*KRAS* p.G12V (6%) *FGFR1* p.E126fs (97%)	*CDKN2A* loss
	2	26.1	NA	NA	NA
	3	36.9	NEG	*ATM* p.I346N (53%)	None
**Fusion and single orange** **(N=6)**	4	76.6	NEG	*TP53 p.*A161D (54%)	*CDKN2A* loss
	5	79.7	POS	*KRAS* p.G12C (74%)	*CDKN2A* loss
	6	87.3	NEG	*TP53* p.G245C (50%); *TP53 p.*E294fs (24%) *BRAF p.*G593F (28%)	*CDKN2A* loss *KRAS* gain *EZH2* gain
	7	82.4	NEG	N/A	NA
	8	50.0	NEG	*EGFR* p.L858R (84%)	*EGFR* gain *CDKN2A* loss
	9	79.7	POS	NA	NA
**Mixed pattern** **(N=3)**	10	30.5	NEG	*TP53 p.*G226R (92%) *FGFR1* E126fs	*CDKN2A* loss
	11	20.7	NEG	*MET* p.T1010I (48%);	*CDKN2A* loss
	12	22.5	NEG	*CTNNB1* p.G34E (61%)	*RET* loss *RB1* loss

NA- insufficient tumor tissue left for IHC or mutation analysis.

The number of cases with positive NGS results was much smaller in the FISH group with single orange pattern (40%). NGS detected *EML4-ALK* fusions in 2 cases with FISH fusion and single orange pattern, and both cases were positive by immunohistochemistry. Two cases in which specific fusion reads crossing the breakpoint were not detected clearly showed elevated 3′/5' *ALK* read ratios, and both cases were also positive by immunohistochemistry. Six of 10 cases (60%) with *ALK* FISH single orange pattern were negative by NGS, and all but 2 cases were negative by immunohistochemistry (Figure 1). A single case that was negative by NGS, but positive by immunohistochemistry also harbored *KRAS* G12C mutation and loss of *CDKN2A*. The second case that was positive by FISH and IHC, did not have a sufficient DNA for additional mutation analysis.

NGS detected no fusions or increased 3′/5' ratio in 3 cases with FISH mixed pattern. All of the cases were negative by ALK immunohistochemistry. The average number of *ALK* FISH positive tumor cells was 24.6% (range 20.7-30.5%). All three cases showed additional mutations and copy number changes of multiple genes as summarized in Table 3.

### Response to crizotinib

**Figure 2:**
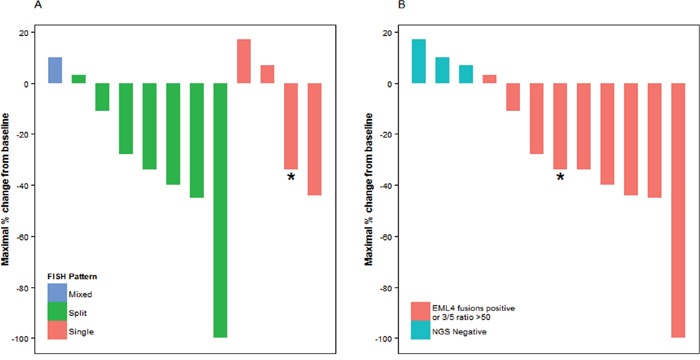
Response to crizotinib and A. ALK FISH patterns, B. NGS ALK fusion detection

Nineteen patients were treated with crizotinib, of whom 12 had at least one radiographic assessment for response. In 7 patients with *ALK* FISH fusion and split signal, the response rate was 57% (one complete response and 3 partial responses), with a stable disease rate of 43% (4 patients) and no patients with progressive disease. In 4 patients with fusion and single orange signal, the response rate was 25% (one partial response), with a 50% stable disease rate (two patients) and a 25% progressive disease rate (one patient) (Figure 2). While there was a numerically higher response rate in the group with FISH split signal, no statistically significant association between responders and FISH patterns was observed (p=0.73). Similarly, there was no statistical association between maximal percent change in tumor size and *ALK* FISH pattern (p=0.24). In contrast, NGS fusion and IHC positive cases were associated with a higher response rate than NGS fusion negative cases (p= 0.016).

## DISCUSSION

Our study demonstrates difficulties and challenges in the assessment and interpretation of the *ALK* rearrangement status. The overall frequency of detection of *ALK* rearrangements by FISH in our study is similar to previous reports. *ALK* FISH assay is currently a gold standard in the assessment of *ALK* gene rearrangements and is highly predictive of response to crizotinib. *ALK* break apart DNA probe is designed to label the 3′ (telomeric) part of the fusion breakpoint with orange signal and the 5′ (centromeric) part with the green signal. The assay is considered to be positive for *ALK* rearrangement if split pattern and/or single orange signal without corresponding green signal are identified in at least 15% of tumor cells, a cut off that was used in all crizotinib studies [1]. There is a relatively small subset of cases with heterogeneity with presence of both positive patterns and these are referred as “mixed patterns”. Similar to study by Camdige et al., our study showed a broad range of positive tumor cells within an overall *ALK* positive tumor [32]. Gao et al. recently challenged the FISH criteria particularly in respect to 5′ deletion manifesting as a “single orange” FISH pattern [31]. In their study, patients with 5′ deletion were older than patients with split signals, although there was no difference in gender and smoking history [31]. We did not find demographic differences between the patients with different FISH patterns in our study. Similar to Gao et al., a group with FISH 5′ deletion showed a higher rate of fusion negative cases by NGS and IHC than the group with a FISH split signal. Furthermore, FISH “mixed group” was negative by both NGS and IHC. Our results suggest that the FISH 5′ deletion and “mixed” patterns may represent false-positive interpretations. One may argue that a single orange pattern is probably related to technical factors such as nuclear sectioning causing loss of the 5′ (green) probe binding site, or simply observer error. Technical errors can't be reliably excluded in a case with a lower percentage of nuclei positive for rearrangement, however in our study a group with single orange pattern and discordant NGS and IHC results showed a large number of positive nuclei (range 50-87%), a finding significantly above the cut off value of 15%. Therefore, we don't believe that this pattern is solely related to technical errors. Furthermore, about 20% of the cases with single orange signal showed discordant results with NGS and IHC. Mutations analysis was also helpful because three cases from this discordant group showed *KRAS*, *BRAF* and *EGFR* L858R mutations suggesting that these mutations are likely to be oncogenic events driving the behavior of these presumably *ALK* positive tumors. Overall, our observations suggest that 5′ deletion pattern represents a heterogenous group of tumors that is more prone to false positive results than cases with split signal. Similarly, mixed cases although limited in number, were all negative by NGS and IHC and showed a lower percentage of *ALK* positive cells. Our study suggest that other approaches such as NGS and IHC may be considered in these two particular groups of cases before decision about ALK-targeted therapy. Pekar-Zlotin et al. recently reported 42.9% sensitivity and 97.7% specificity for ALK FISH when compared to NGS DNA based platform for detection of *ALK* gene rearrangements [10]. They also reported a high concordance between D5F3 antibody and NGS detection of gene rearrangement. As a conclusion, they suggested ALK IHC rather than FISH for the selection of patients for ALK-targeted therapies and NGS to be used in IHC inconclusive cases. Our data also showed a great concordance between IHC and NGS in cases discordant between NGS and FISH. All FISH/NGS discordant cases with FISH split signal showed a lower percentage of *ALK* positive cells and negative ALK IHC, while mutation analysis showed oncogenic mutations of *KRAS* and *ATM* genes. Somewhat similar to our study, Ilie et al. recently reported that the “borderline” number of cells positive for rearrangement is the main reason for discrepancies between FISH and immunohistochemistry results [12]. Of particular importance in this study is that NGS may more reliably select patients most likely to respond to targeted therapy with crizotinib. We identified only two cases that were negative by NGS and positive for *ALK* FISH and IHC. Only one case was available for mutation analysis and showed *KRAS* G12C mutation. This case further supports recently published observations that oncogenic mutations and gene rearrangements in lung carcinoma are not necessarily mutually exclusive [33–35]. This seems to be a rare event and in cases such as this one is hard to tell which genomic event will determine biological and clinical behavior of the tumor. Furthermore, our study supports the idea that lung cancers should be simultaneously tested for a large number of oncogenic mutations, gene rearrangements and probably gene copy number changes since the landscape of genomic abnormalities is complex and may impact tumor response to targeted therapies. Various NGS platforms offer a great advantage of simultaneous detection of numerous genomic abnormalities on a small tumor sample and provide a better understanding of the mutation burden of the tumor which may be of great predictive and prognostic significance. Therefore, NGS approach should be considered as a standard approach to lung cancer testing in a clinical practice.

The main shortcoming of our study is a limited number of cases with treatment data. However, despite small numbers and lack of statistical difference, a group with single orange signal showed mixed responses including cases with and without response to crizotinib, similar to report by Gao et al [31]. In contrast, Camidge et al. reported no correlation between FISH patterns or number of *ALK* positive cell with tumor response to crizotinib treatment [32]. These conflicting observations are very intriguing and should be further prospectively assessed in the larger group of patients.

In summary, our study suggests that *ALK* FISH may not be the most reliable approach for assessment of *ALK* gene rearrangement in lung cancer. Since ALK IHC shows a high concordance with the presence of fusions, it should be considered as an alternative to *ALK* FISH and perhaps as more cost effective initial screening approach. If laboratories decide to run in parallel ALK IHC and ALK FISH than NGS for fusion detection should be considered in cases with discordant results. Furthermore, current practice recommendations do not require detailed reporting of the *ALK* FISH positive patterns, but based on our observations FISH assays tend to show a high rate of false positive results in groups with a single orange signal and with mixed patterns. Therefore, a detailed quantitative reporting of various FISH patterns should be strongly considered as they may suggest false positive results and prompt a pathologist to perform additional IHC or NGS testing. Our observations should be prospectively validated in a larger group of patients.

## MATERIALS AND METHODS

### Patient selection

2116 lung adenocarcinomas and NSCLCs with adenocarcinoma component were tested for *ALK* rearrangements by FISH at the University of Pittsburgh Medical Center from 2009 to 2013. *ALK* rearrangements were detected in 72 cases (4%). Of 72 *ALK* FISH positive adenocarcinoma, 28 were randomly selected based on tissue availability for additional studies. Samples included 16 biopsies (transbronchial, endobronchial, core), 5 resection specimens (wedge, segmentectomy), 5 lymph node mediastinoscopy specimens and 2 pleurectomies.

Clinical information including gender, age, tumor stage, smoking history, and treatment data were obtained from review of patients' electronic medical records. Objective tumor response was determined utilizing Response Evaluation Criteria in Solid Tumors version 1.1, and best overall response was determined for all patients treated with crizotinib who had at least one disease evaluation while on therapy. Maximum tumor shrinkage was calculated utilizing the smallest sum of target lesions after baseline, in reference to baseline tumor measurements. The study was conducted under an exemption approved by the University of Pittsburgh Institutional Review Board (PRO 12070229).

### ALK-immunohistochemistry

Immunohistochemistry for ALK was performed on 4 μm -thick FFPE sections using anti-ALK (D5F3) rabbit monoclonal antibody (Cell Signaling Technology, Danvers, MA) on a BenchMark XT autostainer with the UltraView DAB detection kit (Ventana Medical Systems Inc., Tuscon, AZ). Staining was interpreted as positive if tumor cells showed a moderate or strong multifocal or diffuse expression. All positive cases showed a granular, cytoplasmic pattern.

### *ALK*-FISH

FISH analysis for *ALK* rearrangements was performed as previously reported [36]. In brief, FFPE sections were subjected to FISH analysis by the Vysis ALK Break Apart FISH kit (Abbott Molecular, Abbott Park, IL). The LSI ALK 5′ probe (SpectrumGreen) and the LSI ALK 3′ probe (Spectrum Orange) were applied, hybridized and assessed along with standard controls. At least 60 non-overlapping nuclei were scored for each case and control. The tumors were interpreted as positive for *ALK* rearrangement if split pattern and/or single orange signal without corresponding green signal were identified in at least 15% of tumor cells.

### Next-generation sequencing analysis

#### Nucleic acids isolation

For FFPE tissues, tumor-rich areas (>30-50% of neoplastic cells) were microdissected from three to six 4-μm unstained histologic sections under stereomicroscopic visualization with an Olympus SZ61 microscope (Olympus, Hamburg, Germany). Total nucleic acids were isolated from each target with the DNeasy Blood and Tissue kit on the automated QIAcube (Qiagen, Valencia, CA) instrument according to the manufacturer's instructions. Extracted DNA and RNA were quantitated on the Qubit 2.0 Fluorometer using the dsDNA HS Assay Kit and the RNA HS Assay Kit (Invitrogen, Carlsbad, CA).

#### Detection of gene fusions

For detection of *ALK* fusions, a multiplex amplicon-based targeted NGS panel was used as previously described [29]. In details, this NGS panel allows to detect 169 known gene fusions involving 19 target genes and 94 fusion partners, including *ALK* fusions with the *ATIC, C2orf44, CARS, CLTC, EML4, FN1, KIF5B, KLC1, MSN, NPM1, PPFIBP1, PTPN3, SEC31A, SQSTM1, STRN, TFG, TPM3, TPM4, TRAF1*, and *VCL* genes. The panel also allows detecting novel *ALK* fusions by measuring expression levels between *ALK* extracellular (EC, 5′ end sequencing) and tyrosine kinase (TK, 3′-end sequencing) domains [29].

Gene fusion detection was performed by sequencing of two RNA libraries using 20 ng of RNA (10 ng per amplicon pool) and Ion Total RNA-Seq kit (Life Technologies, Fisher Scientific) according to manufacturer's instructions. Briefly, RNA was reverse transcribed and amplified with the two multiplexed fusion primer pools. Primers were subsequently digested, followed by adapter ligation and emulsion PCR. Sequencing was carried out on an Ion Proton instrument. For detection of *ALK* fusions and *ALK* 3′/5' (TK/EC domains) expression, a custom bioinformatics pipeline was used as previously described [29]. First, raw data in FASTQ format was aligned to a custom reference genome using TMAP [https://github.com/iontorrent/TMAP; accessed Jun 26, 2014] after adapter sequences were removed by cutadapt. FastQC [http://www.bioinformatics.babraham.ac.uk/projects/fastqc/; accessed Jun 26, 2014] was used for quality control of the raw FASTQ data, and alignStats and SAMStat were used to examine the quality of alignment. Visual inspection of the aligned reads for the fusions was performed in Integrative Genomics Viewer (IGV, Broad Institute).

#### Detection of mutations and copy number variations (CNVs)

DNA sequencing was performed using the Ion AmpliSeq™ Cancer Panel (Ion Torrent, Life Technologies, Fisher Scientific) according to the manufacturer's instructions. Briefly, 10 ng of DNA was amplified by PCR using the AmpliSeq™ Cancer Panel Primers pool and Ion AmpliSeq™ Master Mix v2.0. Multiplexed barcoded libraries were enriched by clonal amplification using emulsion PCR on Ion Sphere™ particles (ISPs) (Ion PGM™ Template OT2 200 Kit or Ion PI OT2 200 kit v3) and loaded on an Ion 318™ Chip or Ion P1 Chip (Life Technologies). Massively parallel sequencing was carried out on a Personal Genome Machine™ Sequencer or Ion Proton (Life Technologies, Fisher Scientific). The raw signal data were analyzed using Torrent Suite (version 4.0.1) to generate BAM files after signal processing, base calling adapter trimming and alignment to the reference human genome (hg19). Variants were called with Torrent Suite Variant Caller, and were further analyzed using an internally created software suite. Analysis of copy number variations (CNVs) was performed as previously reported [37].

Sequencing for mutations and CNVs was performed on all cases with discordant results between *ALK* FISH and NGS gene fusion.

### Statistical methodology

Demographic and clinical characteristics were summarized for all 28 subjects, according to *ALK* FISH pattern. Fisher's exact tests were used to test for associations between the *ALK* FISH pattern and gender or smoking history. Kruskal-Wallis tests were used to test for associations between the *ALK* FISH pattern and age or disease stage at the time of diagnosis. Patients were classified as either responders (subjects with complete or partial response) or non-responders (subjects with stable or progressive disease), and statistical association with *ALK* FISH pattern was tested using a Fisher's exact test. Maximal percent change in tumor size was compared between *ALK* FISH patterns with a Kruskal-Wallis test and between NGS fusion positive and negative cases with a Wilcoxon-Mann-Whitney test. Statistical analyses were performed using SAS version 9.4 (SAS Institute Inc., Cary, NC) and RStudio version 0.98.1062 using the ggplot2 package.

## References

[1] Kwak EL, Bang YJ, Camidge DR, Shaw AT, Solomon B, Maki RG, Ou SH, Dezube BJ, Janne PA, Costa DB, Varella-Garcia M, Kim WH, Lynch TJ (2010). Anaplastic lymphoma kinase inhibition in non-small-cell lung cancer. N Engl J Med.

[2] Blackhall FH, Peters S, Bubendorf L, Dafni U, Kerr KM, Hager H, Soltermann A, O'Byrne KJ, Dooms C, Sejda A, Hernandez-Losa J, Marchetti A, Savic S (2014). Prevalence and clinical outcomes for patients with ALK-positive resected stage I to III adenocarcinoma: results from the European Thoracic Oncology Platform Lungscape Project. J Clin Oncol.

[3] Soda M, Choi YL, Enomoto M, Takada S, Yamashita Y, Ishikawa S, Fujiwara S, Watanabe H, Kurashina K, Hatanaka H, Bando M, Ohno S, Ishikawa Y (2007). Identification of the transforming EML4-ALK fusion gene in non-small-cell lung cancer. Nature.

[4] Conde E, Suarez-Gauthier A, Benito A, Garrido P, Garcia-Campelo R, Biscuola M, Paz-Ares L, Hardisson D, de Castro J, Camacho MC, Rodriguez-Abreu D, Abdulkader I, Ramirez J (2014). Accurate identification of ALK positive lung carcinoma patients: novel FDA-cleared automated fluorescence in situ hybridization scanning system and ultrasensitive immunohistochemistry. PLoS One.

[5] Lira ME, Kim TM, Huang D, Deng S, Koh Y, Jang B, Go H, Lee SH, Chung DH, Kim WH, Schoenmakers EF, Choi YL, Park K (2013). Multiplexed gene expression and fusion transcript analysis to detect ALK fusions in lung cancer. J Mol Diagn.

[6] Sholl LM, Weremowicz S, Gray SW, Wong KK, Chirieac LR, Lindeman NI, Hornick JL (2013). Combined use of ALK immunohistochemistry and FISH for optimal detection of ALK-rearranged lung adenocarcinomas. J Thorac Oncol.

[7] Martinez P, Hernandez-Losa J, Montero MA, Cedres S, Castellvi J, Martinez-Marti A, Tallada N, Murtra-Garrell N, Navarro-Mendivill A, Rodriguez-Freixinos V, Canela M, Ramon y Cajal S, Felip E (2013). Fluorescence in situ hybridization and immunohistochemistry as diagnostic methods for ALK positive non-small cell lung cancer patients. PLoS One.

[8] Soda M, Isobe K, Inoue A, Maemondo M, Oizumi S, Fujita Y, Gemma A, Yamashita Y, Ueno T, Takeuchi K, Choi YL, Miyazawa H, Tanaka T (2012). A prospective PCR-based screening for the EML4-ALK oncogene in non-small cell lung cancer. Clin Cancer Res.

[9] Weickhardt AJ, Aisner DL, Franklin WA, Varella-Garcia M, Doebele RC, Camidge DR (2013). Diagnostic assays for identification of anaplastic lymphoma kinase-positive non-small cell lung cancer. Cancer.

[10] Pekar-Zlotin M, Hirsch FR, Soussan-Gutman L, Ilouze M, Dvir A, Boyle T, Wynes M, Miller VA, Lipson D, Palmer GA, Ali SM, Dekel S, Brenner R (2015). Fluorescence in situ hybridization, immunohistochemistry, and next-generation sequencing for detection of EML4-ALK rearrangement in lung cancer. Oncologist.

[11] Cabillic F, Gros A, Dugay F, Begueret H, Mesturoux L, Chiforeanu DC, Dufrenot L, Jauffret V, Dachary D, Corre R, Lespagnol A, Soler G, Dagher J (2014). Parallel FISH and immunohistochemical studies of ALK status in 3244 non-small-cell lung cancers reveal major discordances. J Thorac Oncol.

[12] Ilie MI, Bence C, Hofman V, Long-Mira E, Butori C, Bouhlel L, Lalvee S, Mouroux J, Poudenx M, Otto J, Marquette CH, Hofman P (2015). Discrepancies between FISH and immunohistochemistry for assessment of the ALK status are associated with ALK 'borderline'-positive rearrangements or a high copy number: a potential major issue for anti-ALK therapeutic strategies. Ann Oncol.

[13] Lantuejoul S, Rouquette I, Blons H, Le Stang N, Ilie M, Begueret H, Gregoire V, Hofman P, Gros A, Garcia S, Monhoven N, Devouassoux-Shisheboran M, Mansuet-Lupo A (2015). French multicentric validation of ALK rearrangement diagnostic in 547 lung adenocarcinomas. Eur Respir J.

[14] McLeer-Florin A, Moro-Sibilot D, Melis A, Salameire D, Lefebvre C, Ceccaldi F, de Fraipont F, Brambilla E, Lantuejoul S (2012). Dual IHC and FISH testing for ALK gene rearrangement in lung adenocarcinomas in a routine practice: a French study. J Thorac Oncol.

[15] Minca EC, Portier BP, Wang Z, Lanigan C, Farver CF, Feng Y, Ma PC, Arrossi VA, Pennell NA, Tubbs RR (2013). ALK status testing in non-small cell lung carcinoma: correlation between ultrasensitive IHC and FISH. J Mol Diagn.

[16] Park HS, Lee JK, Kim DW, Kulig K, Kim TM, Lee SH, Jeon YK, Chung DH, Heo DS (2012). Immunohistochemical screening for anaplastic lymphoma kinase (ALK) rearrangement in advanced non-small cell lung cancer patients. Lung Cancer.

[17] Zwaenepoel K, Van Dongen A, Lambin S, Weyn C, Pauwels P (2014). Detection of ALK expression in non-small-cell lung cancer with ALK gene rearrangements–comparison of multiple immunohistochemical methods. Histopathology.

[18] Mino-Kenudson M, Chirieac LR, Law K, Hornick JL, Lindeman N, Mark EJ, Cohen DW, Johnson BE, Janne PA, Iafrate AJ, Rodig SJ (2010). A novel, highly sensitive antibody allows for the routine detection of ALK-rearranged lung adenocarcinomas by standard immunohistochemistry. Clin Cancer Res.

[19] Wynes MW, Sholl LM, Dietel M, Schuuring E, Tsao MS, Yatabe Y, Tubbs RR, Hirsch FR (2014). An international interpretation study using the ALK IHC antibody D5F3 and a sensitive detection kit demonstrates high concordance between ALK IHC and ALK FISH and between evaluators. J Thorac Oncol.

[20] Savic S, Diebold J, Zimmermann AK, Jochum W, Baschiera B, Grieshaber S, Tornillo L, Bisig B, Kerr K, Bubendorf L (2015). Screening for ALK in non-small cell lung carcinomas: 5A4 and D5F3 antibodies perform equally well, but combined use with FISH is recommended. Lung Cancer.

[21] Cutz JC, Craddock KJ, Torlakovic E, Brandao G, Carter RF, Bigras G, Deschenes J, Izevbaye I, Xu Z, Greer W, Yatabe Y, Ionescu D, Karsan A (2014). Canadian anaplastic lymphoma kinase study: a model for multicenter standardization and optimization of ALK testing in lung cancer. J Thorac Oncol.

[22] Sun JM, Choi YL, Won JK, Hirsch FR, Ahn JS, Ahn MJ, Park K (2012). A dramatic response to crizotinib in a non-small-cell lung cancer patient with IHC-positive and FISH-negative ALK. J Thorac Oncol.

[23] Wiesner T, Lee W, Obenauf AC, Ran L, Murali R, Zhang QF, Wong EW, Hu W, Scott SN, Shah RH, Landa I, Button J, Lailler N (2015). Alternative transcription initiation leads to expression of a novel ALK isoform in cancer. Nature.

[24] Lindeman NI, Cagle PT, Beasley MB, Chitale DA, Dacic S, Giaccone G, Jenkins RB, Kwiatkowski DJ, Saldivar JS, Squire J, Thunnissen E, Ladanyi M (2013). Molecular testing guideline for selection of lung cancer patients for EGFR and ALK tyrosine kinase inhibitors: guideline from the College of American Pathologists, International Association for the Study of Lung Cancer, and Association for Molecular Pathology. Arch Pathol Lab Med.

[25] Choi YL, Takeuchi K, Soda M, Inamura K, Togashi Y, Hatano S, Enomoto M, Hamada T, Haruta H, Watanabe H, Kurashina K, Hatanaka H, Ueno T (2008). Identification of novel isoforms of the EML4-ALK transforming gene in non-small cell lung cancer. Cancer Res.

[26] Takeuchi K, Choi YL, Togashi Y, Soda M, Hatano S, Inamura K, Takada S, Ueno T, Yamashita Y, Satoh Y, Okumura S, Nakagawa K, Ishikawa Y (2009). KIF5B-ALK, a novel fusion oncokinase identified by an immunohistochemistry-based diagnostic system for ALK-positive lung cancer. Clin Cancer Res.

[27] Togashi Y, Soda M, Sakata S, Sugawara E, Hatano S, Asaka R, Nakajima T, Mano H, Takeuchi K (2012). KLC1-ALK: a novel fusion in lung cancer identified using a formalin-fixed paraffin-embedded tissue only. PLoS One.

[28] Peled N, Palmer G, Hirsch FR, Wynes MW, Ilouze M, Varella-Garcia M, Soussan-Gutman L, Otto GA, Stephens PJ, Ross JS, Cronin MT, Lipson D, Miller VA (2012). Next-generation sequencing identifies and immunohistochemistry confirms a novel crizotinib-sensitive ALK rearrangement in a patient with metastatic non-small-cell lung cancer. J Thorac Oncol.

[29] Beadling C, Wald AI, Warrick A, Neff TL, Zhong S, Nikiforov YE, Corless CL, Nikiforova MN (2015). A Multiplexed Amplicon Approach for Detecting Gene Fusions by Next-Generation Sequencing. J Mol Diagn.

[30] Abel HJ, Al-Kateb H, Cottrell CE, Bredemeyer AJ, Pritchard CC, Grossmann AH, Wallander ML, Pfeifer JD, Lockwood CM, Duncavage EJ (2014). Detection of gene rearrangements in targeted clinical next-generation sequencing. J Mol Diagn.

[31] Gao X, Sholl LM, Nishino M, Heng JC, Janne PA, Oxnard GR (2015). Clinical Implications of Variant ALK FISH Rearrangement Patterns. J Thorac Oncol.

[32] Camidge DR, Theodoro M, Maxson DA, Skokan M, O'Brien T, Lu X, Doebele RC, Baron AE, Varella-Garcia M (2012). Correlations between the percentage of tumor cells showing an anaplastic lymphoma kinase (ALK) gene rearrangement, ALK signal copy number, and response to crizotinib therapy in ALK fluorescence in situ hybridization-positive nonsmall cell lung cancer. Cancer.

[33] Cai W, Lin D, Wu C, Li X, Zhao C, Zheng L, Chuai S, Fei K, Zhou C, Hirsch FR (2015). Intratumoral Heterogeneity of ALK-Rearranged and ALK/EGFR Coaltered Lung Adenocarcinoma. J Clin Oncol.

[34] Sholl LM, Aisner DL, Varella-Garcia M, Berry LD, Dias-Santagata D, Wistuba II, Chen H, Fujimoto J, Kugler K, Franklin WA, Iafrate AJ, Ladanyi M, Kris MG (2015). Multi-institutional Oncogenic Driver Mutation Analysis in Lung Adenocarcinoma: The Lung Cancer Mutation Consortium Experience. J Thorac Oncol.

[35] Tuononen K, Kero M, Maki-Nevala S, Sarhadi VK, Tikkanen M, Wirtanen T, Ronty M, Knuuttila A, Knuutila S (2014). ALK fusion and its association with other driver gene mutations in Finnish non-small cell lung cancer patients. Genes Chromosomes Cancer.

[36] Li Z, Dacic S, Pantanowitz L, Khalbuss WE, Nikiforova MN, Monaco SE (2014). Correlation of cytomorphology and molecular findings in EGFR+, KRAS+, and ALK+ lung carcinomas. Am J Clin Pathol.

[37] Grasso C, Butler T, Rhodes K, Quist M, Neff TL, Moore S, Tomlins SA, Reinig E, Beadling C, Andersen M, Corless CL (2015). Assessing copy number alterations in targeted, amplicon-based next-generation sequencing data. J Mol Diagn.

